# Dose reduction in whole-body computed tomography of multiple injuries (DoReMI): protocol for a prospective cohort study

**DOI:** 10.1186/1757-7241-22-15

**Published:** 2014-03-03

**Authors:** Dirk Stengel, Caspar Ottersbach, Thomas Kahl, Constanze Nikulka, Claas Güthoff, Thomas Hartel, Sophia Hünnebeck, Axel Ekkernkamp, Sven Mutze

**Affiliations:** 1 Centre for Clinical Research, Unfallkrankenhaus Berlin Trauma Centre, Warener Str. 7, Berlin 12683, Germany; 2 Department of Trauma and Orthopaedic Surgery, Unfallkrankenhaus Berlin Trauma Centre, Warener Str. 7, Berlin 12683, Germany; 3 Department of Radiology, Unfallkrankenhaus Berlin Trauma Centre, Warener Str. 7, Berlin 12683, Germany; 4 Julius Wolff Institute, Charité Medical University Centre, Augustenburger Pl. 1, Berlin 13353, Germany

## Abstract

**Background:**

Single-pass, contrast-enhanced whole body multidetector computed tomography (MDCT) emerged as the diagnostic standard for evaluating patients with major trauma. Modern iterative image algorithms showed high image quality at a much lower radiation dose in the non-trauma setting. This study aims at investigating whether the radiation dose can safely be reduced in trauma patients without compromising the diagnostic accuracy and image quality.

**Methods/Design:**

Prospective observational study with two consecutive cohorts of patients.

*Setting:* A high-volume, academic, supra-regional trauma centre in Germany.

*Study population:* Consecutive male and female patients who 1. had been exposed to a high-velocity trauma mechanism, 2. present with clinical evidence or high suspicion of multiple trauma (predicted Injury Severity Score [ISS] ≥16) and 3. are scheduled for primary MDCT based on the decision of the trauma leader on call.

*Imaging protocols:* In a before/after design, a consecutive series of 500 patients will undergo single-pass, whole-body 128-row multi-detector computed tomography (MDCT) with a standard, as low as possible radiation dose. This will be followed by a consecutive series of 500 patients undergoing an approved ultra-low dose MDCT protocol using an image processing algorithm.

*Data:* Routine administrative data and electronic patient records, as well as digital images stored in a picture archiving and communications system will serve as the primary data source. The protocol was approved by the institutional review board.

*Main outcomes:* (1) incidence of delayed diagnoses, (2) diagnostic accuracy, as correlated to the reference standard of a synopsis of all subsequent clinical, imaging, surgical and autopsy findings, (3) patients’ safety, (4) radiation exposure (e.g. effective dose), (5) subjective image quality (assessed independently radiologists and trauma surgeons on a 100-mm visual analogue scale), (6) objective image quality (e.g., contrast-to-noise ratio).

*Analysis:* Multivariate regression will be employed to adjust and correct the findings for time and cohort effects. An exploratory interim analysis halfway after introduction of low-dose MDCT will be conducted to assess whether this protocol is clearly inferior or superior to the current standard.

**Discussion:**

Although non-experimental, this study will generate first large-scale data on the utility of imaging-enhancing algorithms in whole-body MDCT for major blunt trauma.

**Trial registration:**

Current Controlled Trials ISRCTN74557102.

## Background

According to the most recent injury statistics, every two minutes someone dies of an injury in the European Union [1]. Trauma is, and will remain a significant contributor to years of productive life lost, disability, and health care service costs worldwide [2-4].

The survival and functional prognosis of patients who suffered severe and multiple injuries improved significantly in the industrialized countries [5-8]. Time to appropriate care is an accepted predictor of trauma outcomes, and transport, triage, and work-up intervals must respect this decisive factor. Also, immediately life-threatening injuries and sources of bleeding must be identified (or excluded) with high accuracy [9,10]. Apart from damage-control resuscitation and surgery, the introduction of contrast-enhanced, whole-body, multi-detector computed tomography may be regarded as one of the most important interventions which changed the face of trauma care during the past decade [11-14].

In a recent survey of UK emergency departments, 41 out of 184 (22.3%) respondents indicated that they had a pan-scan policy for major trauma patients, with marked regional variations in availability and reporting times [15]. Data from the German TraumaRegister^D^ suggest that incorporating a routine pan-scan into trauma resuscitation may increase the ratio of observed to expected survivors [16]. A recent analysis confirmed the results in a subsequent cohort and unstable patients [17]. Yet, many injuries to solid organs (e.g., brain, liver, spleen) demarcate only after sufficient resuscitation with crystalloid fluids and blood products. We recently demonstrated in a large cohort of 982 patients that an initial pan-scan is capable to prove but not to exclude injuries to various body regions [18].

Opponents of the whole-body scan have been voicing concerns about patients’ overexposure to radiation with the increasing and often uncritical use of this technology [19-24].

Exposure to radiation for diagnostic purposes is an emerging ethical, medico-legal and public-health issue [25]. It has been estimated that, with the widespread and liberal use of CT, 1.5 - 2.0% of all cancers in the US may now be attributable to the radiation from CT examinations [21]. The median lifetime attributable risk (LAR) of cancer is 4.0 (IQR 0.8 - 11.1) / 1000 for a multiphase abdomen and pelvis CT scan [26].

Modern 64-, 128- and 256-row MDCT scanners are regarded to produce lower dose indexes (CTDI_vol_) than earlier generation single- or 4-row hardware. However, Harrieder et al. recently demonstrated in a retrospective study that, because of enhanced scan lengths, the dose-length-product (DLP) may remain similar or even increase with modern hardware [27]. The authors, however, provided little information on patient demographics (e.g., BMI, ISS) and did not only compare different multi-detector-row technologies but also scanners made by different manufacturers. A comparison of 64-row scanners of four leading companies suggested highest patient mean effective doses (in mSv) produced by the GE LightSpeed VCT (12.7 ± 2.6), followed by the Toshiba Aquilion (11.1 ± 3.3), Philips Brilliance (9.5 ± 0.4) and Siemens Somatom (9.1 ± 1.1) [28].

So-called adaptive statistical iterative algorithms (ASIR), derived from a Bayesian framework, incorporate i. random fluctuations in sinogram measurements [29], ii. non-ideality, iii. different degrees of data credibility, iv. *a priori* information about the distribution of the image space, and other previously unconsidered variables to enhance resolution and reduce artefacts and noise [30].

Distinct technologies like Veo (Model Based Image Reconstruction, GE Healthcare) or IRIS (Iterative Reconstruction in Image Space, Siemens) received FDA approval [31].

Apart from phantom studies and smaller case series, there is currently sparse evidence from head-to-head studies about the effect of ASIR technology on objective and subjective image quality, and exposure to radiation. Exemplarily, Prakash et al. from Boston showed in a cohort of 222 patients who had previously undergone abdominal CT with filtered back projection (FBP) technology and were scheduled for follow-up ASIR scan that CTDL_vol_ and DLP were markedly reduced without compromising image quality [32]. The Prakash group showed similar results in chest CT [33,34]. Other investigators reported similar dose reductions with similar image quality in CT portovenography [35], cerebral CT [36], and abdomino-pelvic CT scans [37].

There is evidence from phantom tests and rigorous clinical evaluations with global clinical collaborators which demonstrate the potential of the FDA-approved iDose system (Philips Healthcare, Eindhoven, The Netherlands) to improve image quality and / or lower radiation dose levels beyond those previously achievable with conventional, routine-dose acquisitions, or FBP reconstructions [38-43].

The combination of BMI-adapted protocols with iterative reconstruction algorithms can reduce radiation exposure to patients and simultaneously improve image quality [38].

Previous non-trauma / elective CT investigations suggest that iterative reconstruction algorithms may maintain image quality at a much lower radiation dose. Yet, large-scale, prospective evidence on the safety and effectiveness of low-dose CT in trauma patients is still sparse. A single retrospective study investigated possible dose reduction at maintained image quality in subjects with multiple injuries [44]. Since results were not adjusted for baseline imbalances (e.g., a 7% difference in the prevalence of comorbidity), conclusions and inferences from this study are difficult to interpret.

Patients with multiple injuries are typically young and thus at a higher risk of CT-induced cancer. Reducing exposure to radiation with constant image quality and diagnostic accuracy in this vulnerable cohort is a key goal of trauma services worldwide, and evidence whether imaging at lower doses of radiation is feasible, safe, and effective is needed. On the other hand, it may be argued that, given the proven benefit of MDCT in lowering trauma-associated mortality, the primary scan must exhaust the full potential of the method and should aim for maximum quality of images to avoid false-negative results, regardless of exposure to radiation.

## Methods/Design

### Design

The Dose Reduction in whole-body computed tomography of Multiple Injuries (DoReMI) investigation is a prospective cohort study employing two large, consecutive cohorts of patients undergoing different approved and established MDCT protocols. All data will be recorded, stored and processed under the conditions of daily practice of trauma care. Gathered data will form the basis for a later interrupted time-series comparison between the effectiveness and safety of the scanning protocols.

### Design history and IRB concerns

The study was originally planned as non-inferiority randomized controlled trial (NI-RCT) with the following hypotheses:

*Null hypothesis* H_0_ notated as:

$$H_{0}:C-T\geq M\;\left(C\;\mathrm{is}\;\mathrm{superior}\;\mathrm{to}\;T\right)$$

where C = image quality in the standard-of-care arm, T = image quality in the experimental (iDose) arm, and M = non-inferiority margin

*Alternative hypothesis* H_A_ expressed as

$$H_{A}:C-T<M\;\left(T\;\mathrm{is}\;\mathrm{not}\;\mathrm{inferior}\;\mathrm{to}\;C\right)$$

In *clinical terms*, the primary hypothesis read as:

In patients with multiple trauma scheduled for single-pass, contrast-enhanced whole-body 128-row MDCT, the iDose algorithm will generate a subjective image quality rated on a visual analogue scale (0 – 100 mm) which is not inferior to standard image production and processing.

The non-inferiority margin (NIM) was set at 1 mm, with a standard deviation of 4 mm (derived from a pilot study of 2 × 20 patients). The sample size for a non-inferiority trial of two means was computed using PASS 11.0 software (NCSS, LLC. Kaysville, Utah, USA). For a fixed NIM of 1 mm, the needed sample size per group was 253 patients to confirm non-inferiority of the experimental compared to the control intervention with a one-sided alpha 0.025 and a power of 80%.

The protocol was submitted to the institutional review board (IRB) of the Charité University Medical Centre, Berlin, Germany, in May 2013. It accounted for 1. the inclusion of unconscious and minor patients in an emergency setting (with distinct processes of informing relatives and deferred informed consent procedures) and 2. varying exposure to radiation for diagnostic purposes as the intervention of interest. Given that MDCT trauma imaging at a lower than usual radiation dose is not the current standard of care in Germany, the institutional review board issued concerns that patients in the experimental group are at risk of missed injuries (not that patients in the standard-of-care group may be exposed to a higher than needed effective dose). Consequently, the initial proposal was rejected. A revised proposal submitted in June 2013 could not solve the IRB’s concerns.

Consequently, the investigators changed their approach to comply with the IRB’s recommendations, and consented on a before/after cohort study. All patients in this study will receive an accepted diagnostic standard of care using approved technologies but will not undergo random allocation to either modality. One may still argue that injuries are acute, unforeseeable events, and there remains a random component in the study set-up.

The ultimate consequence of shifting from an experimental to an observational design was the increase in the sample size to allow for robust multivariate modelling and adjustment of results for confounding.

### Objectives

The following objectives will be addressed:

1. To determine the incidence of missed or delayed diagnoses with either imaging scheme,

2. To define the diagnostic accuracy of either MDCT protocol, based on the synopsis of clinical, imaging, and interventional information gathered until discharge,

3. To evaluate patients’ safety with either protocol,

4. To determine the exposure to radiation and the LAR of cancer with either approach,

5. To evaluate subjective and objective measures of image quality.

### Setting

**Figure 1:**
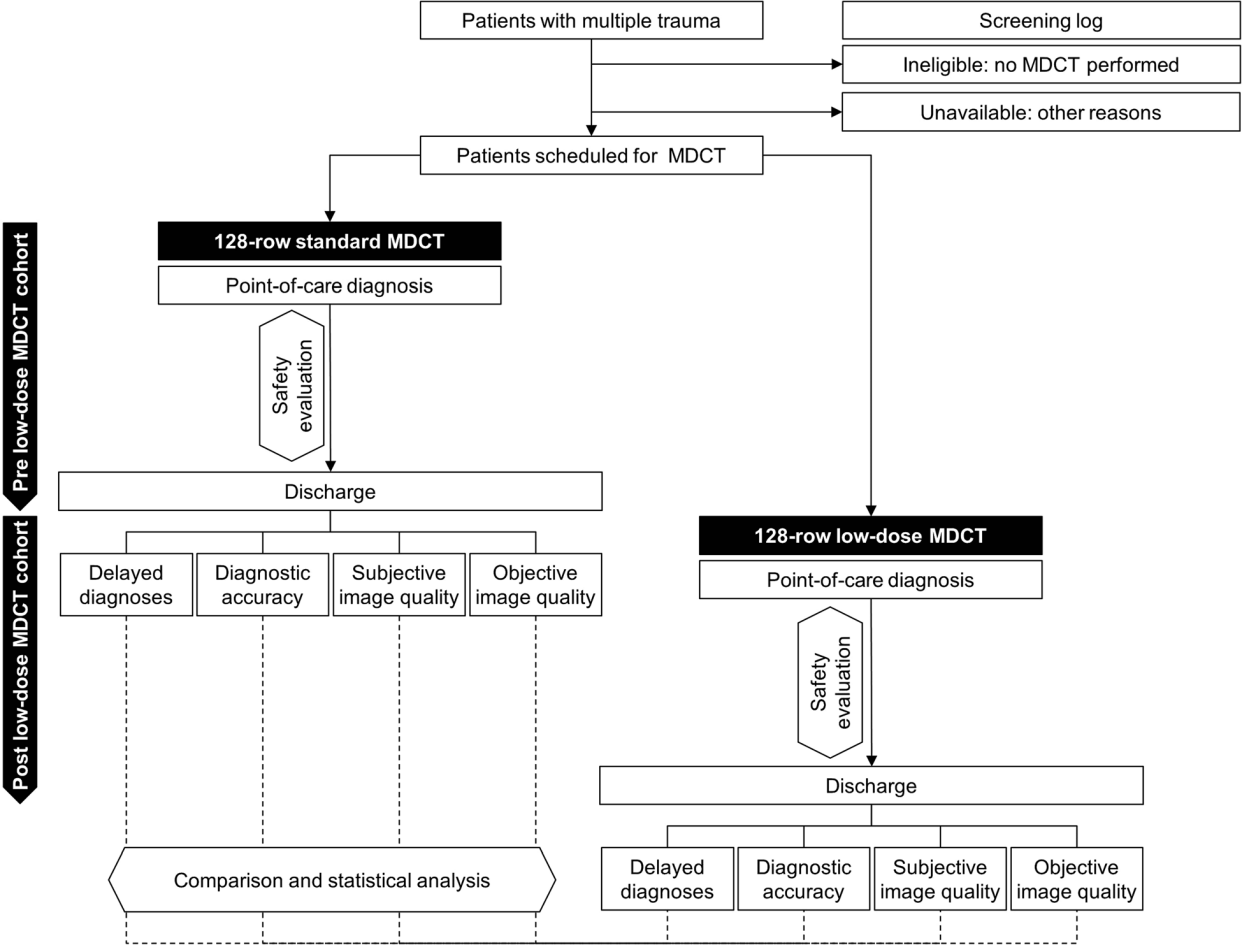
Anticipated study flow.

This is a single-centre non-interventional study using approved and established MDCT imaging protocols for severely injured patients. The study compares a cohort of subjects receiving the standard of care, i.e., single-pass, whole-body 128-MDCT with regular image acquisition and processing, with a subsequent cohort receiving single-pass, whole-body 128-MDCT with a radiation-dose reducing iterative reconstruction method (iDose). Table 1 summarizes key items of both protocols. Figure 1 shows the anticipated study flow.

**Table 1:** Key scanning parameters in the experimental and the control group

	**Standard dose**	**Low dose**
	**128-row MDCT pan-scan**	**128-row MDCT pan-scan + iDose**
Detector z coverage, mm	40	40
kVp	120	120
Exposure, mAs	180	90
iDose Level	off	4 (6)
Recon filter	B	B (C)
CTDI, mGy	11.9	5.9

### Primary outcome

Delayed diagnoses comprise pathological anatomic and / or physiologic findings which demand clinical awareness, or, in case of therapeutically relevant pathological results, should prompt immediate therapeutic action.

We will estimate the incidence of delayed diagnoses based on a synopsis of all subsequent clinical, imaging, intra-operative, and autopsy findings until discharge. We will further distinguish between clinically relevant and all diagnoses. Relevant diagnoses are defined as severe injuries suiting an Abbreviated Injury Scale [AIS] score ≥3 and/or injuries demanding surgical and/or trans-vascular intervention. Non-traumatic incidental findings like tumours or tumour-like lesions demanding specific work-up and/or treatment will be recorded in an exploratory attempt.

### Secondary outcomes

#### Accuracy

Accuracy (or diagnostic efficacy) is a key indicator of performance of an imaging technology. In pan-CT for trauma, there are five variables contributing to overall accuracy:

1. The general technical capability of CT technology to depict a certain pathology (resolution)

2. Trauma-associated, acutely impaired physiological parameters (e.g., hypotension, haemorrhagic shock, centralized circulation) which hamper organ perfusion and/or vessel contrast

3. The interval needed for tissue contusions and hematomas to demark or expand

4. Interpretation of images by the radiologist on call

5. The severity and/or therapeutic consequences of findings

In addition to the incidence of delayed diagnoses, we will compute standard indicators of diagnostic test accuracy (i.e., sensitivity, specificity, positive and negative predictive values, areas under the ROC). Different models will be created for various endpoints (e.g., all missed injuries, therapeutically relevant findings, and others).

#### Safety

Any situation in which the individual patient’s safety is assumed to be compromised will be recorded and evaluated in a cumulative fashion. Safety-relevant events will be entered in the local Critical Incident Reporting System (CIRS), hosted by the Medical Council (Ärztekammer) of Berlin, Germany. Since this is an observational study, we will use common definitions of adverse events (AE), serious adverse events (SAE), and Suspected Unexpected Serious Adverse Reactions (SUSAR), as outlined in ICH Topic E2B (Clinical Safety Data Management: Data Elements for Transmission of Individual Case Safety Reports), and most recently in the amended and updated DIN-ISO 14155 guideline, but do not implement a regular reporting procedure.

#### Radiation dose and estimated LAR of cancer

Dose calculation includes

- CTDI_vol_, scan length, DLP as reported by the scanner (dose report page in PACS), and verified by software CT-Expo, if values are ambiguous or information is missing

- Effective dose (in mSv) calculated by CT-Expo from documented parameters, based on most recent ICRP recommendations (modified tissue-weighting factors)

#### Subjective image quality

Numerous approaches have been proposed to quantify the quality of CT images in the transversal plane, and to generate resource-intensive but clinically required coronal, sagittal, and 3-D reconstruction views.

Basically, there are two different, still complementary concepts to quantify image quality:

1. Subjective evaluation of quality, ideally assessed by two or more independent expert raters in a random, blinded (towards the underlying processing algorithm) fashion, using pre-defined body areas and instruments or scales

2. Objective evaluation, using physical parameters like contrast/signal, noise, and the ratio of both

There is currently no consensus or a reproducible pattern of criteria available to distinguish “good” or “diagnostic” from “unacceptable” or “un-diagnostic” images. In 1999, the European Commission had summarized quality criteria in a guideline (EUR 16262 EN, http://w3.tue.nl/fileadmin/sbd/Documenten/Leergang/BSM/European_Guidelines_Quality_Criteria_Computed_Tomography_Eur_16252.pdf) which had lastly been updated in 2004 (http://www.msct.eu/CT_Quality_Criteria.htm).

Latest updates on the web-platform of the Imaging Performance Assessment of CT Scanners (IMPACT, http://www.impactscan.org/) working group have been made in 2010.

From a statistical point of view, 3- and 5-point ordinal scales have the disadvantage of a non-normal / non-parametric distribution. Although frequently used, the central measure of these scales is not the parametric mean but the geometric mean or median. For sample size calculations and full-scale variance, a visual analogue scale is the better option to determine image quality.

To overcome the limitations with established rating scales, wet set up a pilot study using 2 x 20 sets of images from patients with multiple trauma. The control set consisted of images obtained by a 64-row MDCT scanner (Brilliance 64, Philips, Germany), with scanning parameters published elsewhere [18]. The experimental set comprised images obtained by the 128-row MDCT (Ingenuity Core 128, Philips) scanner to be used in the planned project.

Two independent radiologists blinded to the scanner generation and protocol rated image quality on a 100-mm visual analogue scale, focusing on lung, bone, vessel, fluid/fat and organ tissue contrast.

The arithmetic mean of both raters with the pooled standard deviation s, calculated as

(1) $$s=\sqrt{\frac{\left(n_{1}–1\right)\;S_{1}^{2}+\left(n_{2}–1\right)\;S_{2}^{2}}{n_{1}+n_{2}–2}}$$

was used for quantitative assessment of differences between groups. Here, n_1_ and n_2_ are the number patients / images assessed by rater 1 and 2, and S_1_ and S_2_ are the standard deviations of means.

There were no marked differences in quality ratings between images obtained by either method (Table 2). According to the observed standard deviations, a 1 ± 4 mm difference on the 100-mm VAS appears as the minimally detectable difference between both imaging protocols.

**Table 2:** Summary of the results of the pilot study

**Tissue**	**Pooled mean (SD)**		**Mean difference (95% CI)**
	**64-row**	**128-row**	
Lung	93.90 (1.89)	93.30 (2.03)	0.60 (-0.66 to 1.86)
Bone	93.28 (2.32)	92.15 (3.63)	1.13 (-0.82 to 3.08)
Vessel	91.60 (3.31)	89.90 (4.84)	1.70 (-0.95 to 4.35)
Organ	89.60 (3.92)	85.98 (7.87)	3.62 (-0.36 to 7.60)
Fluid/Fat	92.98 (3.39)	90.78 (4.43)	2.20 (-0.33 to 4.73)

A 100-mm VAS was used for subjective image quality assessment.

#### Objective image quality

Contrast and noise, and the ratio of both, are amongst the most influential indices to describe the objective quality of CT images.

The contrast-to-noise ratio (CNR) is defined as

(2) $$\mathrm{CNR}=\frac{\left|s_{1}–s_{2}\right|}{\sqrt{\frac{\sigma_{1}^{2}+\sigma_{2}^{2}}{2}}}$$

where S_1_ = mean CT number (in HU) in region of interest 1 (ROI_1_) 1, S_2_ = mean HU in ROI_2_, σ_1_ = mean SD of the mean HU in ROI_1_, and σ_2_ mean SD of the mean HU in ROI_2_

Standard ROIs of similar size and position are placed in prominent anatomical structures such as the aorta, liver, other solid organs and spinal muscles. If metal implants are present, then ROIs shall also be placed in the vicinity.

### Patients

This study will include a representative German and European trauma population, meaning that eligible patients had

1. sustained blunt major trauma by a car crash or fall from a height

2. been resuscitated on scene by a multi-professional team of paramedics and emergency physicians, including sedation / general anaesthesia and airway management by oro-tracheal intubation

Thus, the target sample unavoidably includes incompetent (i.e., unconscious and ventilated, probably hemodynamically unstable) patients.

An injury is an unpredictable event that may occur in all age groups, and regardless of other medical conditions. Some patients may also not have reached the legal age of consent at the time of injury.

Patients with the following characteristics are eligible to be enrolled in the planned study:

1. Male and female patients of all ages

2. Suspected or already proven blunt multiple trauma, as indicated by the presence of injuries to ≥2 body regions which, alone or in combination, are life-threatening, or an ISS ≥16

3. *Indication for primary, single-pass whole-body CT by judgment of the trauma leader in charge* and according to red flag criteria outlined in the German Trauma Association’s clinical practice guideline on the management of multiple trauma [45]:

any injury mechanism exposing patients to a high risk of multiple trauma (i.e., road traffic crash with presumed high-energy trauma like extrication or death of a car occupant, pedestrian struck by a vehicle, fall from height)

need for technical rescue

impaired physical or physiological status (i.e., unconsciousness, intubation and ventilation, obvious signs of injury like bruises, haematoma, open wounds or fractures, hemodynamic instability)

suspicion of severe trauma confirmed by paramedics and/or emergency doctors on scene

This study evaluates routine clinical practice and consecutive patients. Since this includes only subjects who undergo primary, single-pass MDCT for clinical indication, those

1. considered unsuitable for CT for any reason (e.g., need for immediate life-saving thoracotomy, laparotomy, or cranial trepanation)

2. with futile resuscitation and / or CPR efforts

3. declared dead on arrival will be missed by this study.

### Recruitment and documentation

Patients will be recruited under routine clinical care conditions, and documentation will mainly comprise data collected for administrative issues or routinely entered in the electronic patient chart. Also, all radiological images will be stored in a PACS for later processing and evaluation.

We will record all patients participating in this study immediately after they underwent the MDCT scan. Informed consent will be gathered by relatives or the next of kin, or individual patients as soon as they are able to consent in using their data for scientific purposes.

This study focuses on delayed and missed injuries, and the accuracy of the primary MDCT scan. Thus, it needs a diagnostic reference or gold standard.

The probably best reference method to which initial MDCT findings can be compared is the clinical and radiological follow-up. If the initial MDCT misses injuries in severely injured patients, it is very likely they will be detected during hospital stay (the same holds true for suspected pathological findings which cannot be confirmed or verified on follow-up imaging).

This study will employ a synopsis of all clinical (including intra-operative) and radiological findings collected until discharge as the diagnostic reference standard. All these items will be traced from routinely available information.

### Sample size considerations and calculations

The sample size is calculated to guarantee a certain precision of a range of estimates, not to achieve a certain power to detect an *a priori* defined effect size.

With 500 eligible cases in either cohort, this study fulfils the following prerequisites:

1. 95% confidence intervals of both risk and mean differences must not exceed the point estimate by more than 5% in either direction

2. multivariate modelling must be possible for five independent covariates (respecting Harrell’s rule)

### Analysis plan

Accepted statistical methods will be used to determine whether the distribution of baseline variables and outcome results are compatible with chance. In general, results will be presented as means, medians, or proportions, and differences in means and proportions, including appropriate measures of distribution and 95% confidence intervals.

We will use established methods to model observational, specifically time-series data, stratified for the different imaging protocols and potential confounding, independent variables. This will include multi-variable generalized linear, random- and mixed-effects regression analyses.

Missing values will be addressed by adequate methods (including multiple imputation techniques).

Multivariate modelling will always be conducted using sound selection, entry, and exclusion of variables, and after testing for model fit and plausibility.

EQUATOR-, GCP-, GEP-, and AMA-conformity will be assured when presenting results. Raw numbers and counts will be provided together with percentages. The STATA 11.0 software package will be employed for all analyses.

### Data management and quality assurance

Data management, including data entry, plausibility checks and query generation will be performed by the local trial coordinating unit (Centre of Clinical Research, Unfallkrankenhaus Berlin).

Subjective quality of images, as assessed on 100-mm VAS for general impression and distinct quality of structures, tissues and sites specified earlier, will be graded by two radiologists in a paper-based fashion. Tick-marks on the VAS will be measured and be entered in an electronic data capture (EDC) system by appointed research assistants at the trial unit.

All further data will be handled and stored electronically for further processing, and processed in the database. Data handling and protection will conducted according to GCP and federal laws of data protection.

All materials pertaining to the investigation will be documented by research staff of the trial unit, sorted and kept in closed archives. The investigator should maintain all source data together with related study documentation for the maximum period of time permitted by the hospital, institution or private practice, but not less than that minimally prescribed by the local authorities after the clinical part of the study has been completed.

## Discussion

This is the first study which assesses the potential value of a dose-reducing MDCT regimen in patients with multiple and severe trauma. The investigators initially planned a RCT which was rejected by the local IRB.

The current protocol aims at balancing regulatory and methodological issues. While the present study (because of its non-experimental design) cannot formally prove that low-dose MDCT is feasible, safe, and evenly accurate compared to normal-dose MDCT in the trauma setting, the cohort size, the setting, and the anticipated characteristics of patient cohorts will guarantee both high internal and external validity of findings. Advanced statistical approaches will allow for quasi-confirmatory inferences. In the absence of large-scales RCTs, this study may provide the best available evidence to decide in favour of one or the other diagnostic approach.

## Competing interests

This is an investigator-initiated study, and no external funding by commercial parties was received for planning this research project. The authors declare they have no competing interest.

## Authors’ contributions

SM and DS conceived the study, and worked together in adapting the protocol according to IRB recommendations and obligations. DS drafted the initial and further protocol versions and developed the initial and subsequent investigation plans. CG is the data manager and statistician of this study, and set up data entry forms and the electronic data capture system. CO is responsible for study administration and regulatory affairs. TK, CN, TH, SH, SM and AE are the site investigators responsible for patient recruitment, data collection, endpoint assessment and judgment of image quality. All authors read and approved the final manuscript.
